# Historical trends in histological composition and cause specific mortality of small intestine tumors based on SEER database analysis

**DOI:** 10.1038/s41598-025-03046-z

**Published:** 2025-05-28

**Authors:** Tianheng Ma, Honggang Wang, Weijie Dai, Peng Shen, Jialing Zhang, Rui Xie

**Affiliations:** Department of Gastroenterology, The Affiliated Huai’an No 1 People’s Hospital of Nanjing Medical University, No.1 Huanghe West Road, Huai’an, 223300 Jiangsu China

**Keywords:** Small intestine, Cause of death, Competing risk, Cancer, Gastrointestinal cancer

## Abstract

Small intestine tumors, though rare, have shown a concerning increase in incidence and mortality in recent years. This study aimed to investigate the historical trends in histological composition and causes of death among patients with small intestine tumors using the SEER database. A total of 18,234 patients diagnosed with primary small intestine cancer from 1992 to 2018 were included in this study. Demographic details, tumor characteristics, treatment modalities, and outcomes were collected. Cause-specific mortality was analyzed using Fine and Gray regression, with non-small intestine-specific deaths considered as competing risks. Small intestine-specific deaths were the leading cause of mortality, with adenocarcinoma and carcinoid tumors being the most common histological types. Heart disease emerged as a significant cause of death following diagnosis, surpassing small intestine-specific deaths after 5–6 years. The study revealed variations in mortality causes across histological subtypes and identified risk factors for small intestine-specific mortality, including age, tumor site, grade, and treatment modalities. The findings highlight the substantial impact of heart disease on the long-term survival of patients with small intestine tumors. This underscores the potential benefits of adopting comprehensive management strategies that integrate oncological and cardiological care to improve survival rates and quality of life. A multidisciplinary approach in oncocardiology may help address the complex needs of these patients and optimize clinical outcomes.

**Supplementary Information:**

The online version contains supplementary material available at 10.1038/s41598-025-03046-z.

## Introduction

Small intestine tumors, although relatively rare among gastrointestinal malignancies, have exhibited a concerning rise in both incidence and mortality in recent years^1^. In 2024, an estimated 12,440 new cases (male: 6,730; female: 5,710) and 2,090 deaths (male: 1,150; female: 940) are projected in the United States^2,3^, compared to just 4,700 new cases and 1,200 deaths in 2000^4^. The most common histological types of small bowel tumors include carcinoid tumor, adenocarcinoma, neuroendocrine tumor, gastrointestinal stromal tumor, and lymphomas^5^. While systemic death risk factors for mortality in small intestine tumors are complex, research on these factors remains limited due to the rarity of this tumor type^6,7^. As the population of small intestine tumor survivors continues to grow, along with their associated systemic risks, a comprehensive understanding of mortality determinants has become increasingly vital for enhancing long-term patient outcomes and quality of life.

Previous studies have linked heart disease to decreased survival rates among patients with small intestinal neuroendocrine tumors^8^. In addition, some studies reported that the risk of heart disease increased in the first 6 months after diagnosis of cancer, with small intestine tumor associated with the highest risk (standardized incidence ratios = 2.88; 95% CI 2.02–3.99)^9^. Despite these findings, the role of heart disease as a cause of death in patients with small intestine tumor remains underexplored.

The Surveillance, Epidemiology, and End Results (SEER) database serves as a comprehensive, population-based resource in the United States, covering approximately 48.0% of the U.S. population. It includes detailed data on patient demographics, primary tumor site, tumor morphology and stage at diagnosis, first course of treatment, and follow-up for vital status^2,10^. In this study based on SEER database, historical trends in histological composition among patients with small intestinal tumors were analyzed, and the alteration in death causes of each histological type was illustrated. Particularly, the assessment of cardiac risk was emphasized and the possible predictors were provided to assess the prognosis of patients with small intestinal tumors.

## Materials and methods

### Data sources

The SEER database is a population-based cancer registry network. The data of SEER research plus data, 13 Registries, Nov 2020 Sub (1992–2018) was queried from SEER database by SEER*Stat software (version 8.3.9.1). This research was performed using publicly available information, without patient and public involvement. Institutional Ethics Committee approval does not apply to the research with publicly available information.

### Study population and definition

**Fig. 1:**
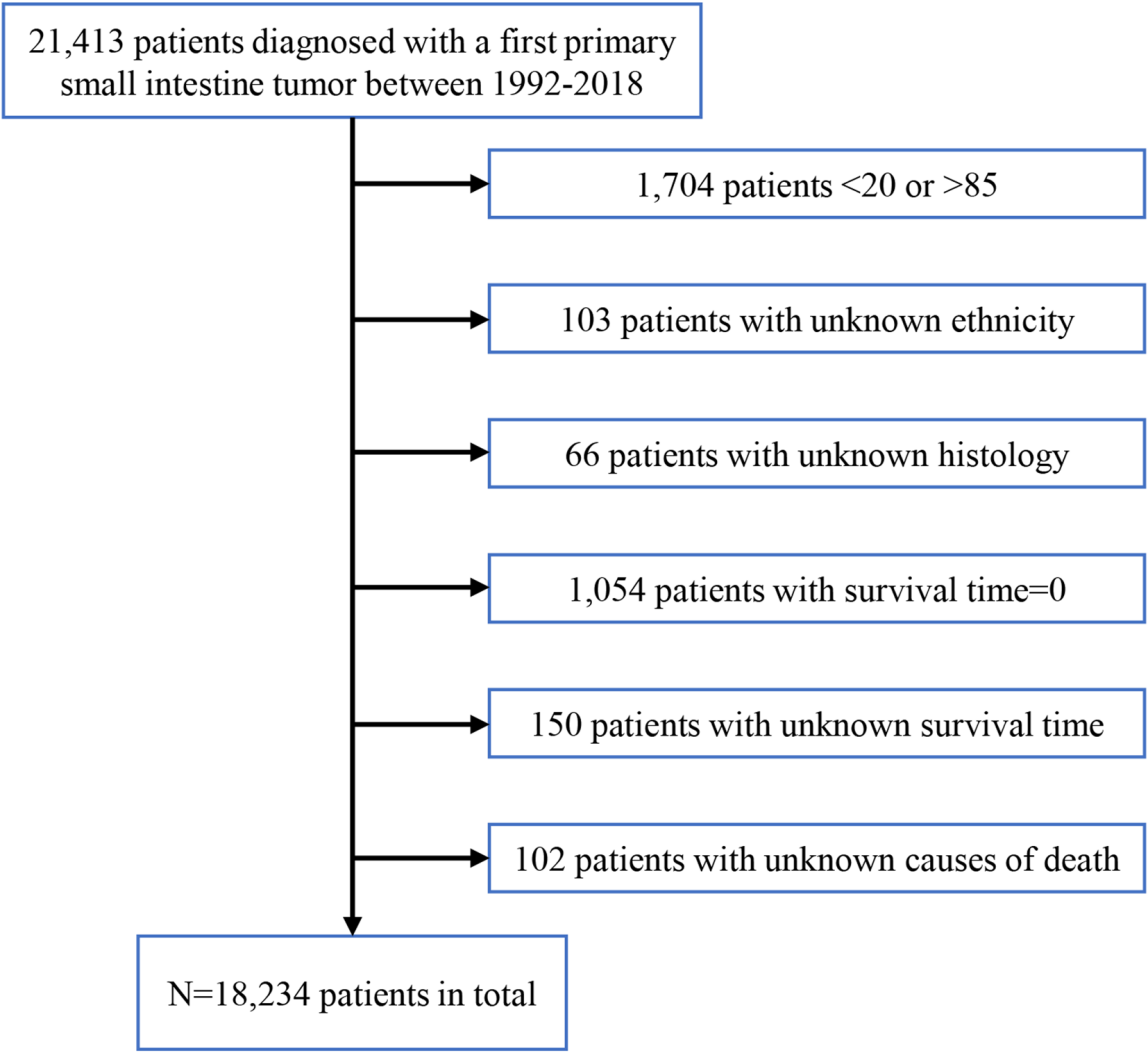
The selection flowchart of eligible patients in SEER database.

The study initially included 21,413 patients diagnosed with primary small intestine cancer between 1 January 1992 and 31 December 2018, as recorded in SEER database. The exclusion criteria included the patients aged < 20 or > 85, those with unknown ethnicity, histology, survival time, or causes of death, as well as those with a survival time of 0. After exclusions, 18,234 patients remained for analysis (Fig. 1). Heart disease specific death was defined as death caused by heart diseases, including acute myocardial infarction, other ischemic heart diseases, or other heart diseases. Small intestine specific death was defined as death caused by small intestine diseases, including carcinoid tumor, adenocarcinoma, neuroendocrine carcinoma, stromal sarcoma, leiomyosarcoma, and others. Digestive tract specific death was defined as death caused by the diseases of esophagus, stomach, colon, and rectum. Due to the limitations of the database, patients with digestive tract specific death might have died from metastases of small intestine tumors, and we were unable to determine whether gastrointestinal-related deaths resulted from metastatic spread of small intestinal tumors or primary gastrointestinal causes. Therefore, this metric should be interpreted with caution.

### Data collection

The demographic data (age, sex, and ethnicity), tumor related data (year of diagnosis, primary site, grade, and metastases), treatment related data (surgery or not, radiation or not, and chemotherapy or not), and outcomes were collected. The age was divided into < 50, 50–59, 60–69, 70–79, and ≥ 80. The primary site included duodenum, jejunum, ileum, meckels diverticulum, overlapping lesion, and small intestine, NOS. The grade included grade I (well differentiated), grade II (moderately differentiated), grade III (poorly differentiated), grade IV (undifferentiated or anaplastic), and unknown grade according to International Classification of Diseases for Oncology, Third Edition (ICD-O-3). The metastases included bone metastases, liver metastases, lung metastases, and brain metastases.

### Statistical analysis

Analyses of demographic and clinical data were performed using SPSS software 25.0 version (IBM Corp, Armonk, NY, USA) and Stata software 16.0 version (Stata Corp, 4905 Lakeway Drive, College Station, USA). The categorical variables were expressed as frequencies and percentages, and the continuous variables were expressed as means ± standard deviations. The demographic and clinical data were analyzed using the chi-square test for categorical variables and analysis of variance for continuous variables. A multivariable Fine and Gray regression analysis was used to analyze the small intestine specific death causes (with non-small intestine specific death as a competing risk). Second order polynomial (quadratic) was employed to fit trends in the small intestine and digestive tract specific death. Hazard ratios (HR) and 95% confidence intervals (CIs) were calculated, and the level of statistical significance was set at *P* < 0.05.

## Result

### Clinical characteristics of the patients with primary small intestine tumors

A total of 21,413 records of patients with primary small intestine tumors between 1992 and 2018 were collected from SEER database, with 18,234 patients meeting inclusion criteria for analysis. For patients with each pathological pattern (i.e., carcinoid tumor, adenocarcinoma, neuroendocrine carcinoma, stromal sarcoma or leiomyosarcoma, and others), most of patients were diagnosed beyond 50 years old. Most patients were diagnosed as the primary small intestine tumors from 2000 to 2018.

It is noteworthy that most pathological pattern of small intestine tumors occurred more possibly in duodenum and ileum, except stromal sarcoma and leiomyosarcoma. Patients with small intestine tumors were less likely to become poorly differentiated, and liver metastases were the most common among the four organs metastases (i.e., bone, liver, lung, and brain). These patients tended to be treated with surgery, while less likely to receive postoperative chemoradiotherapy. The clinical characteristics of the patients with primary small intestine tumors were shown in Table 1.

**Table 1:** Clinical characteristics of the patients with five pathological patterns of primary small intestine tumors.

	Carcinoid Tumor	Adenocarcinoma	Neuroendocrine Carcinoma	Stromal Sarcoma /Leiomyosarcoma	Others	*P*
Age						< 0.001
< 50	1227(14.4%)	740(12.9%)	207(14.2%)	454(23.2%)	110(20.4%)	
≥ 50	1992(23.3%)	1074(18.7%)	366(25.2%)	458(23.5%)	99(18.3%)	
≥ 60	2505(29.4%)	1522(26.5%)	421(28.9%)	518(26.5%)	154(28.5%)	
≥ 70	2138(25.1%)	1706(29.7%)	344(23.6%)	386(19.8%)	127(23.5%)	
≥ 80	671(7.9%)	711(12.4%)	117(8.0%)	137(7.0%)	50(9.3%)	
Sex						0.486
Male	4543(53.2%)	3134(54.5%)	767(52.7%)	1066(54.6%)	295(54.6%)	
Female	3990(46.8%)	2619(45.5%)	688(47.3%)	887(45.4%)	245(45.4%)	
Ethnicity						< 0.001
White	6779(79.4%)	4194(72.9%)	1184(81.4%)	1478(75.7%)	392(72.6%)	
Black	1389(16.3%)	1039(18.1%)	213(14.6%)	148(7.6%)	78(14.4%)	
Other	365(4.3%)	520(9.0%)	58(4.0%)	327(16.7%)	70(13.0%)	
Year of diagnosis						< 0.001
1992–1999	1562(18.3%)	1362(23.7%)	87(6.0%)	384(19.7%)	155(28.7%)	
2000–2018	6971(81.7%)	4391(76.3%)	1368(94.0%)	1569(80.3%)	385(71.3%)	
Primary site						< 0.001
Duodenum	2104(24.7%)	3162(55.0%)	296(20.3%)	372(19.0%)	213(39.4%)	
Jejunum	417(4.9%)	1008(17.5%)	92(6.3%)	478(24.5%)	89(16.5%)	
lleum	3603(42.2%)	740(12.9%)	628(43.2%)	279(14.3%)	107(19.8%)	
Meckels diverticulum	104(1.2%)	5(0.1%)	8(0.5%)	12(0.6%)	2(0.4%)	
Overlapping lesion	87(1.0%)	47(0.8%)	13(0.9%)	34(1.7%)	6(1.1%)	
Small intestine, NOS	2218(26.0%)	791(13.7%)	418(28.7%)	778(39.8%)	123(22.8%)	
Grade						< 0.001
I, well differentiated	2694(31.6%)	389(6.8%)	804(55.3%)	226(11.6%)	22(4.1%)	
II, moderately differentiated	651(7.6%)	2335(40.6%)	254(17.5%)	292(15.0%)	28(5.2%)	
III, poorly differentiated	50(0.6%)	1645(28.6%)	59(4.1%)	89(4.6%)	226(41.9%)	
IV, undifferentiated or anaplastic	16(0.2%)	86(1.5%)	21(1.4%)	171(8.8%)	71(13.1%)	
Unknown	5122(60.0%)	1298(22.6%)	317(21.8%)	1175(60.2%)	193(35.7%)	
Surgery						< 0.001
Yes	6305(73.9%)	3141(54.6%)	1154(79.3%)	1512(77.4%)	315(58.3%)	
No	1106(13.0%)	1622(28.2%)	244(16.8%)	171(8.8%)	115(21.3%)	
Unknown	1122(13.1%)	990(17.2%)	57(3.9%)	270(13.8%)	110(20.4%)	
Radiation						< 0.001
Yes	85(1.0%)	572(9.9%)	29(2.0%)	30(1.5%)	63(11.7%)	
No/unknown	8448(99.0%)	5181(90.1%)	1426(98.0%)	1923(98.5%)	477(88.3%)	
Chemotherapy						< 0.001
Yes	391(4.6%)	2456(42.7%)	148(10.2%)	755(38.7%)	207(38.3%)	
No/unknown	8142(95.4%)	3297(57.3%)	1307(89.8%)	1198(61.3%)	333(61.7%)	
Bone metastases						< 0.001
Yes	28(0.3%)	45(0.8%)	17(1.2%)	3(0.2%)	4(0.7%)	
No	3823(44.8%)	2234(38.8%)	925(63.6%)	783(40.1%)	197(36.5%)	
Unknown	4682(54.9%)	3474(60.4%)	513(35.3%)	1167(59.8%)	339(62.8%)	
Liver metastases						< 0.001
Yes	555(6.5%)	441(7.7%)	264(18.1%)	82(4.2%)	29(5.4%)	
No	3302(38.7%)	1852(32.2%)	684(47.0%)	706(36.1%)	172(31.9%)	
Unknown	4676(54.8%)	3460(60.1%)	507(34.8%)	1165(59.7%)	339(62.8%)	
Lung metastases						< 0.001
Yes	29(0.3%)	143(2.5%)	11(0.8%)	2(0.1%)	4(0.7%)	
No	3824(44.8%)	2133(37.1%)	927(63.7%)	782(40.0%)	196(36.3%)	
Unknown	4680(54.8%)	3477(60.4%)	517(35.5%)	1169(59.9%)	340(63.0%)	
brain metastases						< 0.001
Yes	3(0.0%)	11(0.2%)	4(0.3%)	0(0.0%)	3(0.6%)	
No	3849(45.1%)	2267(39.4%)	937(64.4%)	786(40.2%)	198(36.7%)	
Unknown	4681(54.9%)	3475(60.4%)	514(35.3%)	1167(59.8%)	339(62.8%)	
Outcomes						< 0.001
Dead	3445(40.4%)	4361(75.8%)	566(38.9%)	906(46.4%)	431(79.8%)	
Alive	5088(59.6%)	1392(24.2%)	889(61.1%)	1047(53.6%)	109(20.2%)	

### Constituent ratio changes of small intestinal tumor histological types

**Fig. 2:**
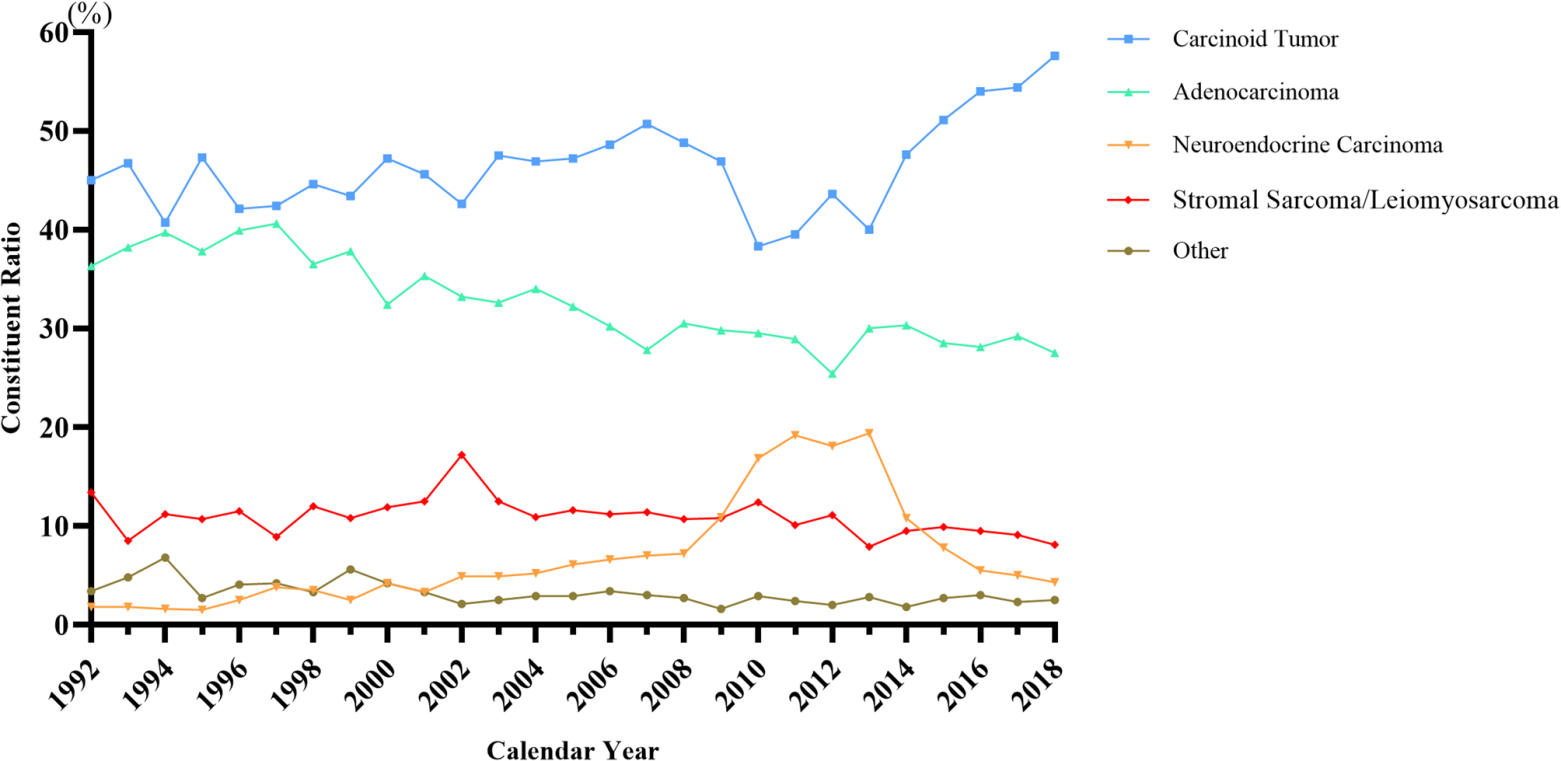
The historical constituent trend for major histological subtypes of small intestinal tumor by successive calendar years (from 1992 to 2018).

The small intestinal carcinoid tumor stably accounted for the largest part among histological types, and the proportion increased to 57.6% in 2018 from 45.0% in 1992. Adenocarcinoma was the second common tumor in small intestine, with slight decrease from the top percentage of 40.6% in 1997 to 27.5% in 2018. Neuroendocrine carcinoma experienced an increase to 19.4% in 2008 to 2013, and then quickly reduced to 4.3% in 2018 thus became the fourth common tumor, while stromal sarcoma or leiomyosarcoma kept at around 10% all along. Other histological types of tumors were rarely found in small intestinal. The change of constituent ratio for each histological type of small intestinal tumors from 1992 to 2018 was shown in Fig. 2; Table 2, and Supplement Tables 1, 2, 3, 4 and 5.

**Table 2:** Cause-specific death in patients with small intestinal tumors.

	Alive	Small Intestine	Digestive Tract	Heart Disease	COPD	Soft Tissue	Pancreas	Miscellaneous Malignant Cancer	Cerebrovascular Disease	Others
1992	37(9.7%)	83(21.8%)	43(11.3%)	36(9.5%)	8(2.1%)	18(4.7%)	9(2.4%)	41(10.8%)	7(1.8%)	98(25.8%)
1993	57(13.0%)	93(21.3%)	48(11.0%)	52(11.9%)	6(1.4%)	15(3.4%)	13(3.0%)	43(9.8%)	11(2.5%)	99(22.7%)
1994	51(13.3%)	76(19.8%)	50(13.1%)	33(8.6%)	4(1.0%)	22(5.7%)	14(3.7%)	30(7.8%)	10(2.6%)	93(24.3%)
1995	52(12.7%)	89(21.7%)	44(10.7%)	44(10.7%)	9(2.2%)	15(3.7%)	12(2.9%)	33(8.0%)	11(2.7%)	101(24.6%)
1996	77(17.3%)	100(22.5%)	49(11.0%)	50(11.3%)	5(1.1%)	18(4.1%)	10(2.3%)	35(7.9%)	7(1.6%)	93(20.9%)
1997	87(17.6%)	93(18.8%)	53(10.7%)	45(9.1%)	6(1.2%)	22(4.4%)	22(4.4%)	51(10.3%)	5(1.0%)	111(22.4%)
1998	84(17.4%)	90(18.7%)	57(11.8%)	41(8.5%)	6(1.2%)	19(3.9%)	19(3.9%)	37(7.7%)	12(2.5%)	117(24.3%)
1999	107(20.6%)	104(20.0%)	61(11.8%)	50(9.6%)	13(2.5%)	8(1.5%)	12(2.3%)	44(8.5%)	9(1.7%)	111(21.4%)
2000	111(23.5%)	81(17.2%)	53(11.2%)	39(8.3%)	5(1.1%)	10(2.1%)	10(2.1%)	45(9.5%)	10(2.1%)	108(22.9%)
2001	128(23.5%)	92(16.9%)	60(11.0%)	40(7.4%)	7(1.3%)	17(3.1%)	11(2.0%)	57(10.5%)	9(1.7%)	123(22.6%)
2002	163(26.5%)	104(16.9%)	67(10.9%)	45(7.3%)	5(0.8%)	12(2.0%)	22(3.6%)	54(8.8%)	10(1.6%)	133(21.6%)
2003	180(28.5%)	94(14.9%)	71(11.2%)	52(8.2%)	4(0.6%)	9(1.4%)	16(2.5%)	50(7.9%)	12(1.9%)	144(22.8%)
2004	212(34.5%)	117(19.1%)	61(9.9%)	34(5.5%)	6(1.0%)	8(1.3%)	14(2.3%)	43(7.0%)	9(1.5%)	110(17.9%)
2005	247(37.7%)	100(15.3%)	59(9.0%)	43(6.6%)	3(0.5%)	12(1.8%)	18(2.7%)	53(8.1%)	10(1.5%)	110(16.8%)
2006	264(40.5%)	98(15.0%)	61(9.4%)	44(6.7%)	4(0.6%)	12(1.8%)	11(1.7%)	45(6.9%)	3(0.5%)	110(16.9%)
2007	273(40.9%)	99(14.8%)	52(7.8%)	44(6.6%)	5(0.7%)	7(1.0%)	14(2.1%)	44(6.6%)	14(2.1%)	116(17.4%)
2008	343(46.7%)	98(13.3%)	46(6.3%)	36(4.9%)	1(0.1%)	7(1.0%)	12(1.6%)	54(7.3%)	7(1.0%)	131(17.8%)
2009	387(50.9%)	104(13.7%)	50(6.6%)	28(3.7%)	2(0.3%)	10(1.3%)	20(2.6%)	46(6.0%)	6(0.8%)	108(14.2%)
2010	459(52.0%)	119(13.5%)	85(9.6%)	33(3.7%)	4(0.5%)	13(1.5%)	17(1.9%)	39(4.4%)	7(0.8%)	106(12.0%)
2011	475(56.2%)	124(14.7%)	60(7.1%)	20(2.4%)	3(0.4%)	5(0.6%)	15(1.8%)	44(5.2%)	2(0.2%)	97(11.5%)
2012	555(60.1%)	95(10.3%)	72(7.8%)	30(3.3%)	5(0.5%)	6(0.7%)	16(1.7%)	35(3.8%)	8(0.9%)	101(10.9%)
2013	567(62.8%)	111(12.3%)	53(5.9%)	21(2.3%)	4(0.4%)	9(1.0%)	19(2.1%)	34(3.8%)	4(0.4%)	81(9.0%)
2014	593(66.1%)	104(11.6%)	56(6.2%)	13(1.4%)	1(0.1%)	5(0.6%)	19(2.1%)	26(2.9%)	4(0.4%)	76(8.5%)
2015	667(67.8%)	113(11.5%)	46(4.7%)	26(2.6%)	3(0.3%)	6(0.6%)	20(2.0%)	32(3.3%)	2(0.2%)	69(7.0%)
2016	722(73.5%)	110(11.2%)	37(3.8%)	13(1.3%)	2(0.2%)	7(0.7%)	8(0.8%)	29(3.0%)	5(0.5%)	49(5.0%)
2017	797(80.2%)	80(8.0%)	32(3.2%)	6(0.6%)	2(0.2%)	1(0.1%)	9(0.9%)	21(2.1%)	4(0.4%)	42(4.2%)
2018	830(89.6%)	39(4.2%)	19(2.1%)	5(0.5%)	2(0.2%)	2(0.2%)	2(0.2%)	8(0.9%)	0(0.0%)	19(2.1%)
Total	8525(46.8%)	2610(14.3%)	1445(7.9%)	923(5.1%)	125(0.7%)	295(1.6%)	384(2.1%)	1073(5.9%)	198(1.1%)	2656(14.6%)

### Cause-specific death in patients with small intestinal tumors

**Fig. 3:**
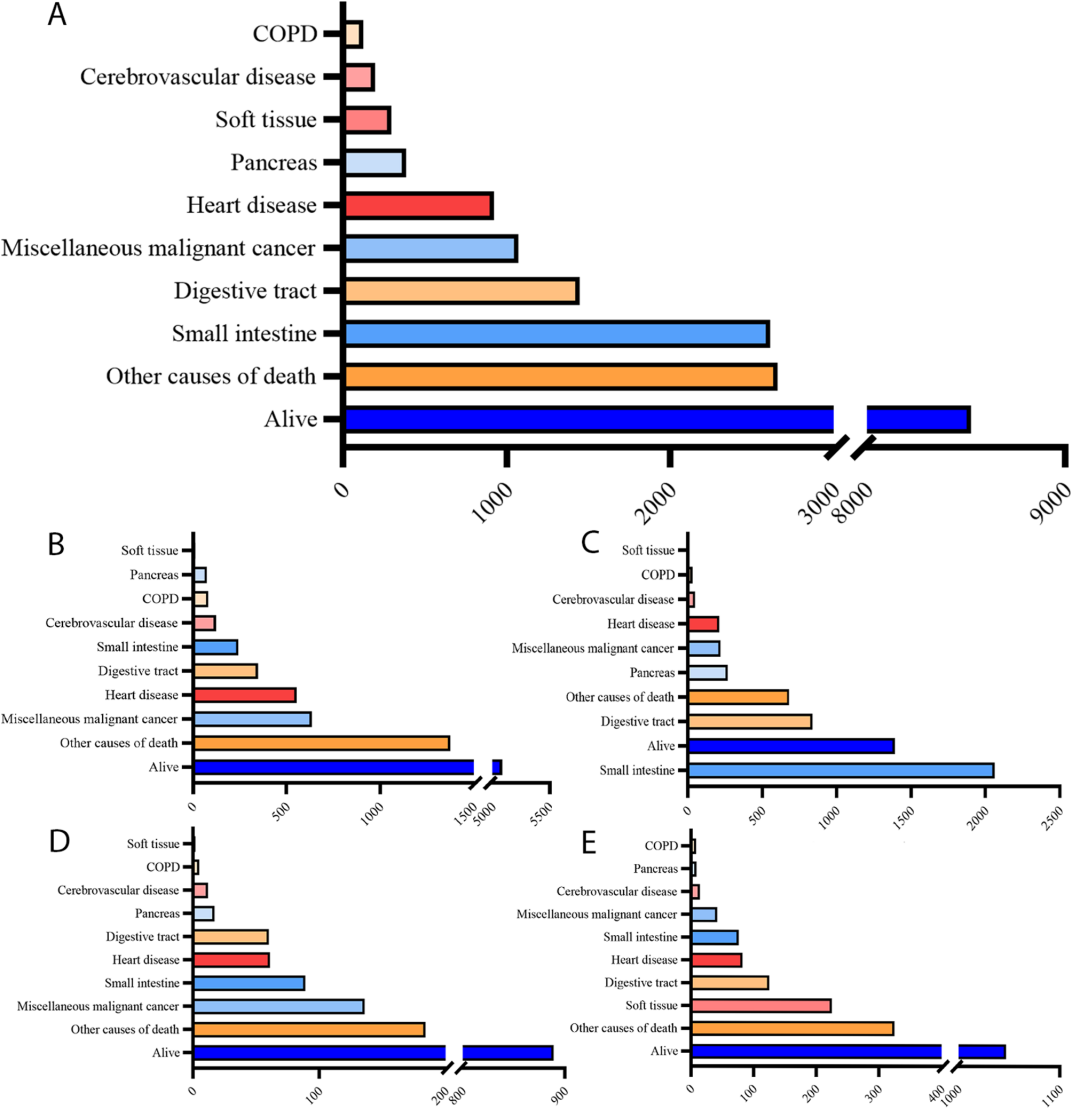
The top ten accumulated death causes of small intestinal tumor in entirety or through its main histological subtypes. (A) The overall top ten accumulated cause-specific death of small intestinal tumor. (B-E) The top ten accumulated cause-specific death separately in (B) carcinoid tumor, (C) adenocarcinoma, (D) neuroendocrine carcinoma and (E) stromal sarcoma or leiomyosarcoma.

In this cohort, a total of 9,709 patients (53.2%) succumbed to small intestinal tumors, and the top 10 accumulated outcomes (Fig. 3A) and their differences in the four histological subtypes (Fig. 3B-E) were analyzed. As a whole, small intestine specific death took the leading status among the known causes, followed by digestive tract, miscellaneous malignant cancer, heart disease, pancreas, soft tissue, cerebrovascular, and COPD. Specifically, small intestinal is the largest cause of death in adenocarcinoma, and separately account for the fifth, the third, and the fifth death causes in carcinoid tumor, neuroendocrine carcinoma, and stromal sarcoma or leiomyosarcoma. Miscellaneous malignant cancer was the second important cause of death both in carcinoid tumor and neuroendocrine tumor. Notably, except of adenocarcinoma, in which heart disease was the sixth specific death cause, digestive tract and heart disease were both former five specific death causes in small intestinal tumors.

**Fig. 4:**
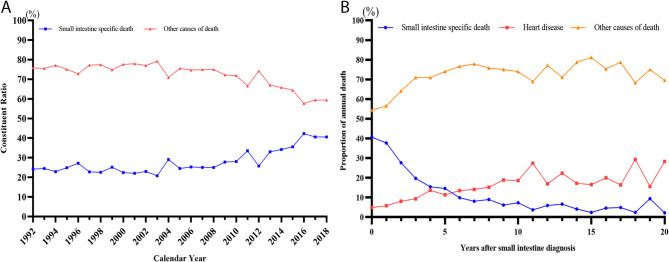
Proportion alteration of death causes in small intestine tumor. (A) Proportion alteration of small intestine specific death and other causes of death during consecutive calenda year (from 1992 to 2018). (B) Proportion alteration of small intestine specific death, heart disease specific death and other causes of death following years after diagnosis.

Moreover, we compared the small intestine specific death with other causes of death in the alterations of their constituent ratio from 1992 to 2018 (Fig. 4A). It turns out that small intestine specific death ascended, yet other causes declined. A slower growth was observed in digestive tract specific death, compared with small intestine specific death (Supplement Fig. 1A). The two trend curves of small intestinal and digestive tract specific death in carcinoid tumors intersected around 2011 (Supplement Fig. 1B), whereas they expanded parallelly in adenocarcinoma (Supplement Fig. 1C).

**Fig. 5:**
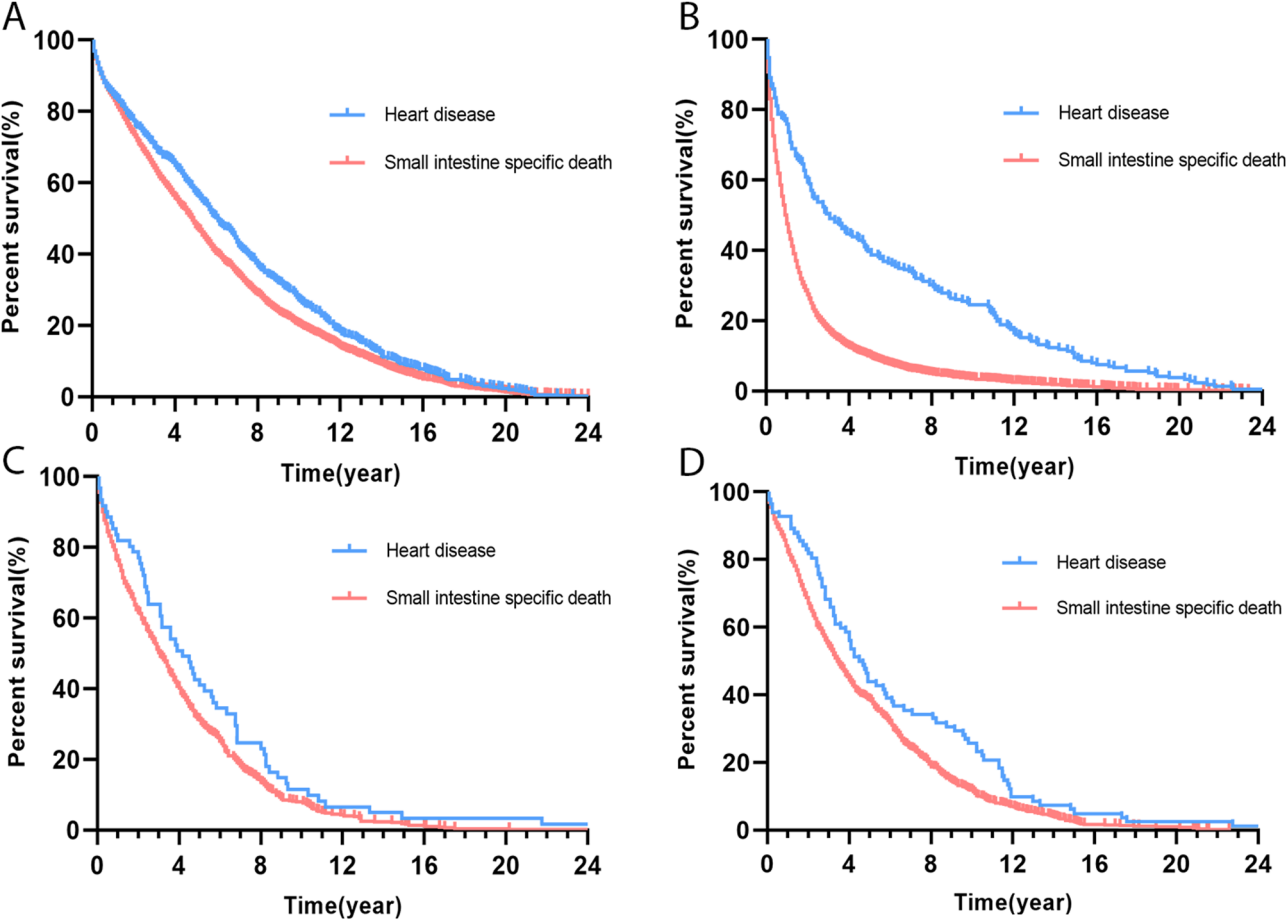
Kaplan-Meier curves of cardiac-specific mortality and small intestine-specific mortality stratified by different pathological types of small intestinal tumors. (**A**) Kaplan-Meier curves of small intestinal tumor patients with carcinoid histology. (**B**) Kaplan-Meier curves of small intestinal tumor patients with adenocarcinoma histology. (**C**) Kaplan-Meier curves of small intestinal tumor patients with neuroendocrine carcinoma histology. (**D**) Kaplan-Meier curves of small intestinal tumor patients with stromal sarcoma or leiomyosarcoma histology.

Furthermore, an elevated cause of heart disease specific death and a decreased cause of small intestine specific death after diagnosis, and the curves intersected at five years after diagnosis (Fig. 4B). Kaplan-Meier (KM) curves revealed distinct patterns: intestinal tumor-related deaths clustered in the initial period, whereas cardiovascular mortality accumulated over time (Fig. 5). Besides, the number of cardiovascular-specific deaths across all age groups has decreased year by year, but the decline in cardiovascular mortality did not show a significant difference between age groups (*P* = 0.232) (Supplement Fig. 2). Subgroup analysis revealed that all-cause mortality (including cardiac death) increased with advancing age (Supplement Table 6).

### Risk factors of small intestinal tumor death

The multivariable Fine and Gray regression analysis was used to analyze the small intestine specific death causes, and the non-small intestine specific death was set as a competing risk (Table 3). It is clearly shown that patients beyond 50 with carcinoid tumor had a higher small intestine specific death, and the HRs were 2.659, 3.252, 2.841, and 2.513 for patients aged ≥ 50, ≥60, ≥ 70, and ≥ 80, respectively. Patients aged ≥ 70 and ≥ 80 with adenocarcinoma or those aged ≥ 80 with neuroendocrine also suffered higher death risk of small intestine. As for the primary site, the risk of small intestine specific death was higher for patients with carcinoid tumors originating from the jejunum, ileum, and Meckel’s diverticulum than those originating from duodenum, with HRs of 2.314, 2.711, and 3.876, respectively. In addition, the HR for patients with adenocarcinoma originating from the ileum was 0.569 compared with those in duodenum. Moreover, patients with poorly differentiated tumor made a larger explosion to small intestine specific death.

**Table 3:** Multivariable fine and Gray regression analysis for small intestine specific death.

	Carcinoid Tumor			Adenocarcinoma			Neuroendocrine Carcinoma			Stromal Sarcoma /Leiomyosarcoma		
	HR	95% CI	*P*	HR	95% CI	*P*	HR	95% CI	*P*	HR	95% CI	*P*
Age												
< 50	1			1			1			1		
≥ 50	**2.659**	**1.454–4.864**	**0.002**	1.055	0.904–1.231	0.500	0.984	0.443–2.185	0.968	1.760	0.873–3.548	0.114
≥ 60	**3.252**	**1.811–5.842**	**< 0.001**	1.064	0.918–1.234	0.409	0.965	0.453–2.058	0.927	1.542	0.779–3.055	0.214
≥ 70	**2.841**	**1.565–5.157**	**0.001**	**1.165**	**1.006–1.349**	**0.041**	1.284	0.595–2.771	0.524	1.344	0.632–2.858	0.443
≥ 80	**2.513**	**1.247–5.067**	**0.010**	**1.261**	**1.053–1.511**	**0.012**	**2.763**	**1.231–6.203**	**0.014**	0.968	0.304–3.086	0.956
Sex												
Male	1			1			1			1		
Female	1.110	0.862–1.429	0.418	0.999	0.915–1.090	0.980	1.328	0.853–2.067	0.209	1.024	0.634–1.656	0.921
Ethnicity												
White	1			1			1			1		
Black	**0.470**	**0.284–0.779**	**0.003**	1.007	0.899–1.127	0.906	0.912	0.479–1.738	0.780	1.332	0.587–3.024	0.492
Other	1.107	0.555–2.207	0.774	0.915	0.783–1.068	0.260	0.794	0.227–2.775	0.718	1.060	0.561–2.003	0.856
Primary site												
Duodenum	1			1			1			1		
Jejunum	**2.314**	**1.09–4.916**	**0.029**	0.967	0.855–1.093	0.591	0.994	0.381–2.597	0.991	0.585	0.284–1.207	0.147
lleum	**2.711**	**1.664–4.414**	**< 0.001**	**0.569**	**0.481–0.672**	**< 0.001**	0.684	0.371–1.263	0.225	0.822	0.396–1.707	0.599
Meckel’s diverticulum	1.623	0.377–6.983	0.516	0.940	0.263–3.359	0.924	——	——	——	——	——	——
Overlapping lesion	**3.876**	**1.412–10.640**	**0.009**	0.902	0.554–1.469	0.679	——	——	——	——	——	——
Small intestine, NOS	**3.621**	**2.255–5.814**	**< 0.001**	**0.732**	**0.633–0.846**	**< 0.001**	1.148	0.655–2.013	0.630	0.813	0.446–1.481	0.498
Grade												
I, well differentiated	1			1			1			1		
II, moderately differentiated	1.589	0.923–2.734	0.095	1.187	0.977–1.442	0.085	1.364	0.688–2.703	0.374	2.357	0.800-6.951	0.120
III, poorly differentiated	**5.064**	**2.289–11.203**	**< 0.001**	**1.591**	**1.305–1.938**	**< 0.001**	**5.278**	**2.433–11.451**	**< 0.001**	2.955	0.798–10.949	0.105
IV, undifferentiated or anaplastic	**7.837**	**2.406–25.528**	**< 0.001**	1.366	0.892–2.090	0.151	**6.176**	**2.064–18.482**	**0.001**	**3.636**	**1.158–11.422**	**0.027**
Unknown	1.198	0.847–1.694	0.307	1.112	0.903–1.370	0.319	**1.905**	**1.013–3.582**	**0.045**	2.074	0.765–5.621	0.152
Surgery												
Yes	1			1			1			1		
No	**2.123**	**1.450–3.107**	**< 0.001**	**2.144**	**1.921–2.392**	**< 0.001**	**1.936**	**1.138–3.291**	**0.015**	1.952	0.904–4.218	0.089
Unknown	1.338	0.976–1.834	0.071	**1.719**	**1.526–1.936**	**< 0.001**	1.721	0.719–4.115	0.223	**4.812**	**2.817–8.219**	**< 0.001**
Radiation												
Yes	1			1			1			1		
No/unknown	**0.424**	**0.200-0.897**	**0.025**	1.055	0.918–1.212	0.454	0.745	0.223–2.487	0.632	**0.283**	**0.122–0.659**	**0.003**
Chemotherapy												
Yes	1			1			1			1		
No/unknown	**0.495**	**0.330–0.743**	**0.001**	**0.602**	**0.546–0.663**	**< 0.001**	**0.385**	**0.215–0.691**	**0.001**	1.152	0.676–1.960	0.603

Furthermore, patients without surgical intervention faced an elevated risk of small intestine specific death (HR = 2.123, 2.144, and 1.936, respectively, for carcinoid tumor, adenocarcinoma, and neuroendocrine carcinoma), while chemotherapy had the opposite effect (HR = 0.495, 0.602, and 0.385, respectively). Similar to chemotherapy, patients with carcinoid tumor, stromal sarcomas or leiomyosarcomas who underwent radiation therapy were at a higher risk of small intestine specific death.

Cox regression analysis was performed to identify risk factors for mortality in patients with small intestinal tumors. The results demonstrated that advanced age, male sex, poor tumor differentiation, lack of surgical intervention, and presence of metastasis were all risk factors for death (Supplement Table 7).

## Discussion

This study, based on data from the SEER database, explored the historical trend of histological composition in patients with small intestinal tumors and analyzed changes in cause-specific mortality for each histological type. Our findings confirm that small intestinal tumors pose a significant threat to the patients’ lives. Furthermore, we utilized the competing risk model to reveal risk factors associated with small intestine specific death, including age, primary site, tumor grade, surgery, radiation therapy, and chemotherapy. In addition, the importance of assessing cardiac risks was emphasized in patients with small intestinal tumors, particularly in the years following diagnosis, which emerged as a critical factor influencing outcomes.

Abou et al. identified 3,280 patients with small intestinal carcinoid tumor from the Explorys database, reporting a prevalence was 9.2/100,000, with a higher incidence in men and elderly (aged > 65)^11^. It was in line with our study. Moreover, the small intestinal carcinoid tumor was the most common tumor, and increased during 1992 and 2018 according our study. Patients with small intestinal carcinoid tumors could have a relatively favorable prognosis, with 59.6% (5088/8533) of patients surviving from the disease. Only 2.9% (243/8533) of patients suffered from small intestine specific death. Moreover, the small intestinal adenocarcinoma tended to be the second most common tumor, with a slight decline over the past 30 years. In France, adenocarcinoma was the most common histological type (38%), followed by neuroendocrine tumors (35%), lymphoma (15%), and sarcoma (12%)^12^. Their study indicated the historical predominance of adenocarcinoma, which could largely be attributed to regional difference in predisposing diseases and patients’ age structure^13^. In addition, the small intestine represents 75% of the length in the alimentary tract, while only 2% of malignant gastrointestinal tumors were detected^14^. However, based on a significant delay in diagnosis of small intestine adenocarcinoma compared with stomach and colon cancer, it was almost incurable at operation.

According to WHO 5th edition classification of neuroendocrine neoplasms (NENs)^15^, carcinoid tumors refer to well-differentiated NENs of grade G1 to G3, which are equivalent to neuroendocrine tumors (NETs). In contrast, neuroendocrine carcinomas (NECs) were defined as poorly differentiated NENs with a higher grade. In our study, neuroendocrine carcinomas remained the last common tumor in small intestinal during the periods 1992–2008 and 2014–2018. Notably, from 2008 to 2014, there was a transient peak in the incidence of NECs, accompanied by a decline in carcinoid tumors, likely due to the change in classification. Stromal sarcoma and leiomyosarcoma were relatively rare among small intestinal tumors. It was reported that small bowel brought 31.8% of gastrointestinal stromal tumor^16^, which composed 0.1 to 3% of all malignancies in the gastrointestinal tract^17^. In addition, the leiomyosarcoma was account for an estimated 2–3% of small bowel cancers^18^. Due to their benign majority, slow progress, and nonspecific symptoms of stromal sarcoma and leiomyosarcoma, clinical diagnosis mainly depends on conventional CT and MRI, as well as incidental finding^17,19^, which leads to delay in diagnosis.

Besides, we observed an increasing ratio of small intestine specific death, with a relative decline in other causes, while the overall proportion of total deaths gradually decreased. According to multivariable Fine and Gray regression analysis, the small intestine specific death was attributed to older age (≥ 50 for carcinoid; ≥70 for adenocarcinoma), specific tumor site (jejunum and ileum for carcinoid; ileum for adenocarcinoma), poorer differentiation (Grade III and IV), and lack of surgery for patients with carcinoid and adenocarcinoma, while lack of chemoradiotherapy was associated with increased mortality from other causes. Furthermore, tumor grade, surgery, and chemoradiotherapy also played an important role in the small intestine specific death for patients with neuroendocrine carcinoma and stromal sarcoma/leiomyosarcoma. Zandee et al. reported that most small intestinal neuroendocrine tumors arise from embryonic midgut (jejunum and ileum), where live more intestinal chromaffin cells, to emit serotonin^20^. Zhang et al. reported that the median overall and progression-free survival rates for patients with advanced small bowel adenocarcinomas who underwent surgical treatment were 22.0 and 13.0 months, respectively^21^. Onkendi et al. revealed that surgical treatments could improve the prognosis of patients with small bowel adenocarcinomas (five-year overall survival was 37–38%)^22^. These were consistent with our study that surgery is a protective factor for small intestine specific death. However, the chemoradiotherapy showed an equivocal or nonsignificant prognostic benefit based on the current studies. Our multivariate analysis indicates that patients received chemoradiotherapy had borderline significance (*P* = 0.075)^23^. Overman et al. further reported that chemoradiotherapy could not improve the disease-free survival (*P* = 0.15)^24^. Our study reported that the chemoradiotherapy could improve survival from no-small intestine specific death, it also contributed to an increase in small intestine specific death. This may be due to the higher degree of malignancy of small intestinal tumors in patients requiring chemoradiotherapy, which may lead to more small intestine specific death. Furthermore, according to our knowledge, this is the first study to explore the risk factors of small intestine specific death, rather than total causes of death.

Interestingly, small intestine specific death gradually decreased, while heart diseases specific death increased following the diagnosis of small intestinal tumors. Heart diseases specific death surpassed small intestine specific death at 5–6 years after the time of diagnosis, with 28.3% of patients dying from small intestine specific causes and 2.2% from heart disease after 20 years. In view of the relative well long-term survival rate of small intestinal tumors^5,25,26^, basic cardiac diseases play an important role in elderly patients to evaluate the prognosis of small intestinal tumors. Furthermore, the impact of carcinoid heart disease on individuals with small intestine neuroendocrine tumors cannot be overlooked. Roughly 40%~50% of individuals with carcinoid syndrome have carcinoid heart disease^27^. Small intestinal carcinoids frequently secrete excessive serotonin (5-hydroxytryptamine, 5-HT) that escapes hepatic metabolism due to venous drainage bypassing the portal system. Chronic exposure to elevated circulating serotonin induces fibrotic changes in cardiac valves through activation of 5-HT2B receptors on valvular interstitial cells. This pathognomonic carcinoid heart disease typically manifests as right-sided valvular lesions (tricuspid regurgitation and pulmonary stenosis) due to first-pass exposure. The resultant valvular dysfunction progresses insidiously, ultimately causing right heart failure - a major contributor to mortality in these patients^28^. While our registry-based study lacked direct serotonin measurements or echocardiographic validation, this well-established pathophysiology may partially explain the heightened cardiac mortality observed in our carcinoid subgroup. Future studies incorporating biomarker assessments and cardiac imaging would help quantify this mechanism’s contribution.

Our study has several limitations that should be acknowledged. First, due to the limitations of the database, we were unable to determine whether gastrointestinal-related deaths resulted from metastatic spread of small intestinal tumors or primary gastrointestinal causes. Second, the SEER database lacks detailed patient comorbidities (e.g., underlying cardiac history) and treatment specifics (e.g., chemotherapy regimens), which may influence survival outcomes and cause-of-death attribution. Without these data, we could not adjust for potential confounding effects of competing risks or treatment heterogeneity. Third, tumor molecular characteristics (e.g., mutational profiles, biomarkers) are unavailable in SEER, limiting our ability to assess their prognostic impact. Forth, cause-of-death coding in SEER relies on death certificates, which may occasionally misclassify underlying mortality causes. Therefore, this metric should be interpreted with caution. Despite these constraints, our analysis provides valuable population-level insights into the epidemiological and survival patterns of small intestinal sarcomas. Future studies integrating clinical, molecular, and treatment data are warranted to validate and extend our findings.

## Conclusion

In conclusion, this study illustrated the historical change of cause specific death in small intestinal tumors, and identified risk factors for heart disease specific death. The management of heart disease in cancer patients has always been a complex problem, which is seriously related to the survival of small intestinal tumors. Therefore, multidisciplinary cooperation is needed to promote the development of oncocardiology in order to improve the quality of life and overall survival time of patients with small intestinal tumors.

## Electronic supplementary material

Below is the link to the electronic supplementary material.

Supplementary Material 1

Supplementary Material 2

Supplementary Material 3

Supplementary Material 4

Supplementary Material 5

Supplementary Material 6

Supplementary Material 7

Supplementary Material 8

Supplementary Material 9

Supplementary Material 10

## Data Availability

Reasonable requests for data and materials will be considered and should be made in writing to the corresponding author.
