# Supplementary material for: Historical trends in histological composition and cause specific mortality of small intestine tumors based on SEER database analysis
Source: Sci Rep. 2025 May 28;15:18628. doi: 10.1038/s41598-025-03046-z (PMC12120026; doi:10.1038/s41598-025-03046-z)
Supplement: Supplementary file 1 — Supplementary Material 1 [file 41598_2025_3046_MOESM1_ESM.docx]

|  | Carcinoid Tumor | Adenocarcinoma | Neuroendocrine Carcinoma | Stromal Sarcoma /Leiomyosarcoma | Others |
| --- | --- | --- | --- | --- | --- |
| 1992 | 171(45.0%) | 138(36.3%) | 7(1.8%) | 51(13.4%) | 13(3.4%) |
| 1993 | 204(46.7%) | 167(38.2%) | 8(1.8%) | 37(8.5%) | 21(4.8%) |
| 1994 | 156(40.7%) | 152(39.7%) | 6(1.6%) | 43(11.2%) | 26(6.8%) |
| 1995 | 194(47.3%) | 155(37.8%) | 6(1.5%) | 44(10.7%) | 11(2.7%) |
| 1996 | 187(42.1%) | 177(39.9%) | 11(2.5%) | 51(11.5%) | 18(4.1%) |
| 1997 | 210(42.4%) | 201(40.6%) | 19(3.8%) | 44(8.9%) | 21(4.2%) |
| 1998 | 215(44.6%) | 176(36.5%) | 17(3.5%) | 58(12.0%) | 16(3.3%) |
| 1999 | 225(43.4%) | 196(37.8%) | 13(2.5%) | 56(10.8%) | 29(5.6%) |
| 2000 | 223(47.2%) | 153(32.4%) | 20(4.2%) | 56(11.9%) | 20(4.2%) |
| 2001 | 248(45.6%) | 192(35.3%) | 18(3.3%) | 68(12.5%) | 18(3.3%) |
| 2002 | 262(42.6%) | 204(33.2%) | 30(4.9%) | 106(17.2%) | 13(2.1%) |
| 2003 | 300(47.5%) | 206(32.6%) | 31(4.9%) | 79(12.5%) | 16(2.5%) |
| 2004 | 288(46.9%) | 209(34.0%) | 32(5.2%) | 67(10.9%) | 18(2.9%) |
| 2005 | 309(47.2%) | 211(32.2%) | 40(6.1%) | 76(11.6%) | 19(2.9%) |
| 2006 | 317(48.6%) | 197(30.2%) | 43(6.6%) | 73(11.2%) | 22(3.4%) |
| 2007 | 339(50.7%) | 186(27.8%) | 47(7.0%) | 76(11.4%) | 20(3.0%) |
| 2008 | 359(48.8%) | 224(30.5%) | 53(7.2%) | 79(10.7%) | 20(2.7%) |
| 2009 | 357(46.9%) | 227(29.8%) | 83(10.9%) | 82(10.8%) | 12(1.6%) |
| 2010 | 338(38.3%) | 260(29.5%) | 149(16.9%) | 109(12.4%) | 26(2.9%) |
| 2011 | 334(39.5%) | 244(28.9%) | 162(19.2%) | 85(10.1%) | 20(2.4%) |
| 2012 | 402(43.6%) | 234(25.4%) | 167(18.1%) | 102(11.1%) | 18(2.0%) |
| 2013 | 361(40.0%) | 271(30.0%) | 175(19.4%) | 71(7.9%) | 25(2.8%) |
| 2014 | 427(47.6%) | 272(30.3%) | 97(10.8%) | 85(9.5%) | 16(1.8%) |
| 2015 | 503(51.1%) | 280(28.5%) | 77(7.8%) | 97(9.9%) | 27(2.7%) |
| 2016 | 530(54.0%) | 276(28.1%) | 54(5.5%) | 93(9.5%) | 29(3.0%) |
| 2017 | 541(54.4%) | 290(29.2%) | 50(5.0%) | 90(9.1%) | 23(2.3%) |
| 2018 | 533(57.6%) | 255(27.5%) | 40(4.3%) | 75(8.1%) | 23(2.5%) |
| Total | 8533(46.8%) | 5753(31.6%) | 1455(8.0%) | 1953(10.7%) | 540(3.0%) |
