# Supplementary material for: Historical trends in histological composition and cause specific mortality of small intestine tumors based on SEER database analysis
Source: Sci Rep. 2025 May 28;15:18628. doi: 10.1038/s41598-025-03046-z (PMC12120026; doi:10.1038/s41598-025-03046-z)
Supplement: Supplementary file 2 — Supplementary Material 2 [file 41598_2025_3046_MOESM2_ESM.docx]

Supplement Table 2

|  | Alive | Small Intestine | Digestive Tract | Heart Disease | COPD | Soft Tissue | Pancreas | Miscellaneous Malignant Cancer | Cerebrovascular Disease | Others |
| --- | --- | --- | --- | --- | --- | --- | --- | --- | --- | --- |
| 1992 | 18(10.5%) | 11(6.4%) | 21(12.3%) | 24(14.0%) | 6(3.5%) | 0(0.0%) | 0(0.0%) | 31(18.1%) | 2(1.2%) | 58(33.9%) |
| 1993 | 34(16.7%) | 8(3.9%) | 23(11.3%) | 27(13.2%) | 3(1.5%) | 0(0.0%) | 2(1.0%) | 32(15.7%) | 8(3.9%) | 67(32.8%) |
| 1994 | 31(19.9%) | 5(3.2%) | 14(9.0%) | 18(11.5%) | 1(0.6%) | 1(0.6%) | 2(1.3%) | 21(13.5%) | 5(3.2%) | 58(37.2%) |
| 1995 | 30(15.5%) | 12(6.2%) | 20(10.3%) | 32(16.5%) | 8(4.1%) | 0(0.0%) | 2(1.0%) | 24(12.4%) | 8(4.1%) | 58(29.9%) |
| 1996 | 44(23.5%) | 13(7.0%) | 17(9.1%) | 24(12.8%) | 4(2.1%) | 1(0.5%) | 4(2.1%) | 30(16.0%) | 3(1.6%) | 47(25.1%) |
| 1997 | 55(26.2%) | 9(4.3%) | 16(7.6%) | 30(14.3%) | 3(1.4%) | 1(0.5%) | 3(1.4%) | 29(13.8%) | 1(0.5%) | 63(30.0%) |
| 1998 | 53(24.7%) | 11(5.1%) | 16(7.4%) | 23(10.7%) | 3(1.4%) | 1(0.5%) | 3(1.4%) | 26(12.1%) | 8(3.7%) | 71(33.0%) |
| 1999 | 68(30.2%) | 10(4.4%) | 21(9.3%) | 28(12.4%) | 9(4.0%) | 0(0.0%) | 3(1.3%) | 25(11.1%) | 7(3.1%) | 54(24.0%) |
| 2000 | 74(33.2%) | 10(4.5%) | 18(8.1%) | 21(9.4%) | 4(1.8%) | 1(0.4%) | 2(0.9%) | 34(15.2%) | 6(2.7%) | 53(23.8%) |
| 2001 | 69(27.8%) | 9(3.6%) | 14(5.6%) | 32(12.9%) | 5(2.0%) | 1(0.4%) | 5(2.0%) | 34(13.7%) | 8(3.2%) | 71(28.6%) |
| 2002 | 90(34.4%) | 18(6.9%) | 10(3.8%) | 23(8.8%) | 5(1.9%) | 1(0.4%) | 4(1.5%) | 41(15.6%) | 8(3.1%) | 62(23.7%) |
| 2003 | 116(38.7%) | 11(3.7%) | 19(6.3%) | 33(11.0%) | 4(1.3%) | 1(0.3%) | 5(1.7%) | 32(10.7%) | 6(2.0%) | 73(24.3%) |
| 2004 | 125(43.4%) | 16(5.6%) | 19(6.6%) | 22(7.6%) | 3(1.0%) | 0(0.0%) | 2(0.7%) | 27(9.4%) | 6(2.1%) | 68(23.6%) |
| 2005 | 145(46.9%) | 7(2.3%) | 18(5.8%) | 35(11.3%) | 1(0.3%) | 1(0.3%) | 5(1.6%) | 37(12.0%) | 6(1.9%) | 54(17.5%) |
| 2006 | 172(54.3%) | 10(3.2%) | 14(4.4%) | 27(8.5%) | 2(0.6%) | 0(0.0%) | 3(0.9%) | 24(7.6%) | 1(0.3%) | 64(20.2%) |
| 2007 | 185(54.6%) | 13(3.8%) | 12(3.5%) | 29(8.6%) | 3(0.9%) | 0(0.0%) | 2(0.6%) | 33(9.7%) | 8(2.4%) | 54(15.9%) |
| 2008 | 206(57.4%) | 10(2.8%) | 11(3.1%) | 22(6.1%) | 1(0.3%) | 1(0.3%) | 3(0.8%) | 33(9.2%) | 4(1.1%) | 68(18.9%) |
| 2009 | 233(65.3%) | 8(2.2%) | 7(2.0%) | 15(4.2%) | 1(0.3%) | 1(0.3%) | 3(0.8%) | 24(6.7%) | 5(1.4%) | 60(16.8%) |
| 2010 | 223(66.0%) | 13(3.8%) | 11(3.3%) | 20(5.9%) | 3(0.9%) | 0(0.0%) | 7(2.1%) | 10(3.0%) | 6(1.8%) | 45(13.3%) |
| 2011 | 254(76.0%) | 6(1.8%) | 9(2.7%) | 8(2.4%) | 1(0.3%) | 0(0.0%) | 5(1.5%) | 14(4.2%) | 1(0.3%) | 36(10.8%) |
| 2012 | 304(75.6%) | 5(1.2%) | 10(2.5%) | 15(3.7%) | 3(0.7%) | 1(0.2%) | 1(0.2%) | 15(3.7%) | 5(1.2%) | 43(10.7%) |
| 2013 | 294(81.4%) | 6(1.7%) | 5(1.4%) | 10(2.8%) | 1(0.3%) | 1(0.3%) | 1(0.3%) | 13(3.6%) | 2(0.6%) | 28(7.8%) |
| 2014 | 358(83.8%) | 8(1.9%) | 6(1.4%) | 8(1.9%) | 1(0.2%) | 0(0.0%) | 1(0.2%) | 11(2.6%) | 2(0.5%) | 32(7.5%) |
| 2015 | 421(83.7%) | 5(1.0%) | 5(1.0%) | 15(3.0%) | 2(0.4%) | 0(0.0%) | 3(0.6%) | 11(2.2%) | 2(0.4%) | 39(7.8%) |
| 2016 | 466(87.9%) | 4(0.8%) | 4(0.8%) | 9(1.7%) | 1(0.2%) | 0(0.0%) | 1(0.2%) | 15(2.8%) | 3(0.6%) | 27(5.1%) |
| 2017 | 501(92.6%) | 2(0.4%) | 6(1.1%) | 3(0.6%) | 1(0.2%) | 0(0.0%) | 2(0.4%) | 6(1.1%) | 2(0.4%) | 18(3.3%) |
| 2018 | 519(97.4%) | 3(0.6%) | 0(0.0%) | 0(0.0%) | 1(0.2%) | 0(0.0%) | 0(0.0%) | 4(0.8%) | 0(0.0%) | 6(1.1%) |
| Total | 5088(59.6%) | 243(2.8%) | 346(4.1%) | 553(6.5%) | 80(0.9%) | 13(0.2%) | 74(0.9%) | 636(7.5%) | 123(1.4%) | 1377(16.1%) |
