# Supplementary material for: Historical trends in histological composition and cause specific mortality of small intestine tumors based on SEER database analysis
Source: Sci Rep. 2025 May 28;15:18628. doi: 10.1038/s41598-025-03046-z (PMC12120026; doi:10.1038/s41598-025-03046-z)
Supplement: Supplementary file 3 — Supplementary Material 3 [file 41598_2025_3046_MOESM3_ESM.docx]

Supplement Table 3

|  | Alive | Small Intestine | Digestive Tract | Heart Disease | COPD | Soft Tissue | Pancreas | Miscellaneous Malignant Cancer | Cerebrovascular Disease | Others |
| --- | --- | --- | --- | --- | --- | --- | --- | --- | --- | --- |
| 1992 | 12(8.7%) | 56(40.6%) | 17(12.3%) | 7(5.1%) | 1(0.7%) | 0(0.0%) | 8(5.8%) | 7(5.1%) | 4(2.9%) | 26(18.8%) |
| 1993 | 14(8.4%) | 75(44.9%) | 19(11.4%) | 21(12.6%) | 2(1.2%) | 1(0.6%) | 10(6.0%) | 7(4.2%) | 2(1.2%) | 16(9.6%) |
| 1994 | 12(7.9%) | 60(39.5%) | 29(19.1%) | 8(5.3%) | 2(1.3%) | 1(0.7%) | 7(4.6%) | 4(2.6%) | 4(2.6%) | 25(16.4%) |
| 1995 | 14(9.0%) | 67(43.2%) | 19(12.3%) | 10(6.5%) | 1(0.6%) | 0(0.0%) | 8(5.2%) | 7(4.5%) | 2(1.3%) | 27(17.4%) |
| 1996 | 16(9.0%) | 72(40.7%) | 22(12.4%) | 20(11.3%) | 1(0.6%) | 1(0.6%) | 6(3.4%) | 5(2.8%) | 3(1.7%) | 31(17.5%) |
| 1997 | 18(9.0%) | 77(38.3%) | 29(14.4%) | 10(5.0%) | 3(1.5%) | 2(1.0%) | 16(8.0%) | 12(6.0%) | 4(2.0%) | 30(14.9%) |
| 1998 | 18(10.2%) | 62(35.2%) | 31(17.6%) | 9(5.1%) | 3(1.7%) | 0(0.0%) | 15(8.5%) | 7(4.0%) | 4(2.3%) | 27(15.3%) |
| 1999 | 13(6.6%) | 77(39.3%) | 31(15.8%) | 16(8.2%) | 3(1.5%) | 0(0.0%) | 7(3.6%) | 12(6.1%) | 2(1.0%) | 35(17.9%) |
| 2000 | 13(8.5%) | 63(41.2%) | 21(13.7%) | 13(8.5%) | 1(0.7%) | 1(0.7%) | 7(4.6%) | 7(4.6%) | 2(1.3%) | 25(16.3%) |
| 2001 | 34(17.7%) | 71(37.0%) | 34(17.7%) | 5(2.6%) | 1(0.5%) | 0(0.0%) | 6(3.1%) | 14(7.3%) | 0(0.0%) | 27(14.1%) |
| 2002 | 22(10.8%) | 75(36.8%) | 36(17.6%) | 12(5.9%) | 0(0.0%) | 0(0.0%) | 16(7.8%) | 8(3.9%) | 2(1.0%) | 33(16.2%) |
| 2003 | 29(14.1%) | 71(34.5%) | 40(19.4%) | 9(4.4%) | 0(0.0%) | 0(0.0%) | 9(4.4%) | 10(4.9%) | 2(1.0%) | 36(17.5%) |
| 2004 | 32(15.3%) | 93(44.5%) | 28(13.4%) | 5(2.4%) | 3(1.4%) | 0(0.0%) | 12(5.7%) | 9(4.3%) | 2(1.0%) | 25(12.0%) |
| 2005 | 46(21.8%) | 82(38.9%) | 25(11.8%) | 3(1.4%) | 1(0.5%) | 0(0.0%) | 11(5.2%) | 8(3.8%) | 2(0.9%) | 33(15.6%) |
| 2006 | 39(19.8%) | 76(38.6%) | 34(17.3%) | 9(4.6%) | 1(0.5%) | 1(0.5%) | 7(3.6%) | 6(3.0%) | 1(0.5%) | 23(11.7%) |
| 2007 | 37(19.9%) | 70(37.6%) | 32(17.2%) | 6(3.2%) | 0(0.0%) | 0(0.0%) | 12(6.5%) | 5(2.7%) | 3(1.6%) | 21(11.3%) |
| 2008 | 60(26.8%) | 82(36.6%) | 26(11.6%) | 9(4.0%) | 0(0.0%) | 0(0.0%) | 9(4.0%) | 12(5.4%) | 1(0.4%) | 25(11.2%) |
| 2009 | 56(24.7%) | 84(37.0%) | 32(14.1%) | 6(2.6%) | 0(0.0%) | 0(0.0%) | 14(6.2%) | 10(4.4%) | 0(0.0%) | 25(11.0%) |
| 2010 | 66(25.4%) | 91(35.0%) | 56(21.5%) | 4(1.5%) | 0(0.0%) | 0(0.0%) | 7(2.7%) | 12(4.6%) | 0(0.0%) | 24(9.2%) |
| 2011 | 60(24.6%) | 101(41.4%) | 36(14.8%) | 4(1.6%) | 0(0.0%) | 0(0.0%) | 9(3.7%) | 8(3.3%) | 1(0.4%) | 25(10.2%) |
| 2012 | 56(23.9%) | 85(36.3%) | 48(20.5%) | 4(1.7%) | 0(0.0%) | 0(0.0%) | 11(4.7%) | 7(3.0%) | 1(0.4%) | 22(9.4%) |
| 2013 | 72(26.6%) | 94(34.7%) | 41(15.1%) | 4(1.5%) | 2(0.7%) | 0(0.0%) | 17(6.3%) | 10(3.7%) | 1(0.4%) | 30(11.1%) |
| 2014 | 91(33.5%) | 86(31.6%) | 49(18.0%) | 2(0.7%) | 0(0.0%) | 1(0.4%) | 14(5.1%) | 6(2.2%) | 1(0.4%) | 22(8.1%) |
| 2015 | 97(34.6%) | 97(34.6%) | 32(11.4%) | 7(2.5%) | 1(0.4%) | 0(0.0%) | 14(5.0%) | 10(3.6%) | 0(0.0%) | 22(7.9%) |
| 2016 | 119(43.1%) | 90(32.6%) | 30(10.9%) | 4(1.4%) | 1(0.4%) | 1(0.4%) | 6(2.2%) | 6(2.2%) | 1(0.4%) | 18(6.5%) |
| 2017 | 158(54.5%) | 71(24.5%) | 23(7.9%) | 3(1.0%) | 1(0.3%) | 0(0.0%) | 7(2.4%) | 9(3.1%) | 2(0.7%) | 16(5.5%) |
| 2018 | 188(73.7%) | 33(12.9%) | 17(6.7%) | 2(0.8%) | 1(0.4%) | 0(0.0%) | 2(0.8%) | 1(0.4%) | 0(0.0%) | 11(4.3%) |
| Total | 1392(24.2%) | 2061(35.8%) | 837(14.5%) | 212(3.7%) | 29(0.5%) | 9(0.2%) | 267(4.6%) | 219(3.8%) | 47(0.8%) | 680(11.8%) |
