# Supplementary material for: Historical trends in histological composition and cause specific mortality of small intestine tumors based on SEER database analysis
Source: Sci Rep. 2025 May 28;15:18628. doi: 10.1038/s41598-025-03046-z (PMC12120026; doi:10.1038/s41598-025-03046-z)
Supplement: Supplementary file 4 — Supplementary Material 4 [file 41598_2025_3046_MOESM4_ESM.docx]

Supplement Table 4

|  | Alive | Small Intestine | Digestive Tract | Heart Disease | COPD | Soft Tissue | Pancreas | Miscellaneous Malignant Cancer | Cerebrovascular Disease | Others |
| --- | --- | --- | --- | --- | --- | --- | --- | --- | --- | --- |
| 1992 | 0(0.0%) | 1(14.3%) | 1(14.3%) | 1(14.3%) | 0(0.0%) | 0(0.0%) | 0(0.0%) | 1(14.3%) | 0(0.0%) | 3(42.9%) |
| 1993 | 2(25.0%) | 2(25.0%) | 0(0.0%) | 0(0.0%) | 1(12.5%) | 0(0.0%) | 0(0.0%) | 2(25.0%) | 0(0.0%) | 1(12.5%) |
| 1994 | 1(16.7%) | 1(16.7%) | 0(0.0%) | 2(33.3%) | 0(0.0%) | 0(0.0%) | 0(0.0%) | 2(33.3%) | 0(0.0%) | 0(0.0%) |
| 1995 | 0(0.0%) | 1(16.7%) | 0(0.0%) | 1(16.7%) | 0(0.0%) | 0(0.0%) | 0(0.0%) | 1(16.7%) | 1(16.7%) | 2(33.3%) |
| 1996 | 0(0.0%) | 2(18.2%) | 2(18.2%) | 2(18.2%) | 0(0.0%) | 0(0.0%) | 0(0.0%) | 0(0.0%) | 0(0.0%) | 5(45.5%) |
| 1997 | 3(15.8%) | 1(5.3%) | 1(5.3%) | 0(0.0%) | 0(0.0%) | 0(0.0%) | 1(5.3%) | 7(36.8%) | 0(0.0%) | 6(31.6%) |
| 1998 | 2(11.8%) | 2(11.8%) | 2(11.8%) | 3(17.6%) | 0(0.0%) | 1(5.9%) | 0(0.0%) | 1(5.9%) | 0(0.0%) | 6(35.3%) |
| 1999 | 3(23.1%) | 2(15.4%) | 0(0.0%) | 2(15.4%) | 0(0.0%) | 0(0.0%) | 1(7.7%) | 3(23.1%) | 0(0.0%) | 2(15.4%) |
| 2000 | 1(5.0%) | 4(20.0%) | 2(10.0%) | 0(0.0%) | 0(0.0%) | 0(0.0%) | 0(0.0%) | 4(20.0%) | 1(5.0%) | 8(40.0%) |
| 2001 | 7(38.9%) | 2(11.1%) | 3(16.7%) | 0(0.0%) | 0(0.0%) | 0(0.0%) | 0(0.0%) | 3(16.7%) | 0(0.0%) | 3(16.7%) |
| 2002 | 8(26.7%) | 4(13.3%) | 7(23.3%) | 3(10.0%) | 0(0.0%) | 0(0.0%) | 1(3.3%) | 2(6.7%) | 0(0.0%) | 5(16.7%) |
| 2003 | 9(29.0%) | 4(12.9%) | 0(0.0%) | 3(9.7%) | 0(0.0%) | 0(0.0%) | 1(3.2%) | 4(12.9%) | 3(9.7%) | 7(22.6%) |
| 2004 | 21(65.6%) | 2(6.3%) | 2(6.3%) | 2(6.3%) | 0(0.0%) | 0(0.0%) | 0(0.0%) | 4(12.5%) | 0(0.0%) | 1(3.1%) |
| 2005 | 18(45.0%) | 6(15.0%) | 4(10.0%) | 3(7.5%) | 0(0.0%) | 0(0.0%) | 1(2.5%) | 4(10.0%) | 1(2.5%) | 3(7.5%) |
| 2006 | 15(34.9%) | 4(9.3%) | 3(7.0%) | 4(9.3%) | 0(0.0%) | 0(0.0%) | 0(0.0%) | 11(25.6%) | 0(0.0%) | 6(14.0%) |
| 2007 | 13(27.7%) | 7(14.9%) | 3(6.4%) | 3(6.4%) | 2(4.3%) | 0(0.0%) | 0(0.0%) | 4(8.5%) | 1(2.1%) | 14(29.8%) |
| 2008 | 29(54.7%) | 3(5.7%) | 1(1.9%) | 3(5.7%) | 0(0.0%) | 0(0.0%) | 0(0.0%) | 4(7.5%) | 0(0.0%) | 13(24.5%) |
| 2009 | 47(56.6%) | 7(8.4%) | 6(7.2%) | 4(4.8%) | 0(0.0%) | 0(0.0%) | 3(3.6%) | 8(9.6%) | 1(1.2%) | 7(8.4%) |
| 2010 | 93(62.4%) | 7(4.7%) | 5(3.4%) | 7(4.7%) | 0(0.0%) | 0(0.0%) | 2(1.3%) | 13(8.7%) | 0(0.0%) | 22(14.8%) |
| 2011 | 98(60.5%) | 9(5.6%) | 7(4.3%) | 6(3.7%) | 1(0.6%) | 1(0.6%) | 0(0.0%) | 16(9.9%) | 0(0.0%) | 24(14.8%) |
| 2012 | 122(73.1%) | 1(0.6%) | 5(3.0%) | 6(3.6%) | 1(0.6%) | 0(0.0%) | 3(1.8%) | 8(4.8%) | 2(1.2%) | 19(11.4%) |
| 2013 | 139(79.4%) | 4(2.3%) | 4(2.3%) | 5(2.9%) | 0(0.0%) | 0(0.0%) | 0(0.0%) | 11(6.3%) | 1(0.6%) | 11(6.3%) |
| 2014 | 73(75.3%) | 2(2.1%) | 0(0.0%) | 0(0.0%) | 0(0.0%) | 0(0.0%) | 3(3.1%) | 8(8.2%) | 1(1.0%) | 10(10.3%) |
| 2015 | 59(76.6%) | 4(5.2%) | 1(1.3%) | 1(1.3%) | 0(0.0%) | 0(0.0%) | 1(1.3%) | 8(10.4%) | 0(0.0%) | 3(3.9%) |
| 2016 | 43(79.6%) | 5(9.3%) | 1(1.9%) | 0(0.0%) | 0(0.0%) | 0(0.0%) | 0(0.0%) | 4(7.4%) | 0(0.0%) | 1(1.9%) |
| 2017 | 45(90.0%) | 2(4.0%) | 0(0.0%) | 0(0.0%) | 0(0.0%) | 0(0.0%) | 0(0.0%) | 2(4.0%) | 0(0.0%) | 1(2.0%) |
| 2018 | 38(95.0%) | 0(0.0%) | 0(0.0%) | 0(0.0%) | 0(0.0%) | 0(0.0%) | 0(0.0%) | 1(2.5%) | 0(0.0%) | 1(2.5%) |
| Total | 889(61.1%) | 89(6.1%) | 60(4.1%) | 61(4.2%) | 5(0.3%) | 2(0.1%) | 17(1.2%) | 136(9.3%) | 12(0.8%) | 184(12.6%) |
