# Supplementary material for: Historical trends in histological composition and cause specific mortality of small intestine tumors based on SEER database analysis
Source: Sci Rep. 2025 May 28;15:18628. doi: 10.1038/s41598-025-03046-z (PMC12120026; doi:10.1038/s41598-025-03046-z)
Supplement: Supplementary file 5 — Supplementary Material 5 [file 41598_2025_3046_MOESM5_ESM.docx]

Supplement Table 5

|  | Alive | Small Intestine | Digestive Tract | Heart Disease | COPD | Soft Tissue | Pancreas | Miscellaneous Malignant Cancer | Cerebrovascular Disease | Others |
| --- | --- | --- | --- | --- | --- | --- | --- | --- | --- | --- |
| 1992 | 5(9.8%) | 11(21.6%) | 3(5.9%) | 3(5.9%) | 0(0.0%) | 16(31.4%) | 0(0.0%) | 2(3.9%) | 1(2%) | 10(19.6%) |
| 1993 | 5(13.5%) | 3(8.1%) | 1(2.7%) | 3(8.1%) | 0(0.0%) | 11(29.7%) | 0(0.0%) | 1(2.7%) | 1(2.7%) | 12(32.4%) |
| 1994 | 5(11.6%) | 4(9.3%) | 3(7.0%) | 3(7.0%) | 1(2.3%) | 16(37.2%) | 1(2.3%) | 3(7.0%) | 1(2.3%) | 6(14.0%) |
| 1995 | 8(18.2%) | 5(11.4%) | 3(6.8%) | 1(2.3%) | 0(0.0%) | 14(31.8%) | 0(0.0%) | 1(2.3%) | 0(0.0%) | 12(27.3%) |
| 1996 | 12(23.5%) | 8(15.7%) | 5(9.8%) | 3(5.9%) | 0(0.0%) | 14(27.5%) | 0(0.0%) | 0(0.0%) | 1(2.0%) | 8(15.7%) |
| 1997 | 9(20.5%) | 3(6.8%) | 4(9.1%) | 4(9.1%) | 0(0.0%) | 15(34.1%) | 1(2.3%) | 1(2.3%) | 0(0.0%) | 7(15.9%) |
| 1998 | 9(15.5%) | 10(17.2%) | 7(12.1%) | 5(8.6%) | 0(0.0%) | 14(24.1%) | 0(0.0%) | 3(5.2%) | 0(0.0%) | 10(17.2%) |
| 1999 | 22(39.3%) | 4(7.1%) | 4(7.1%) | 4(7.1%) | 1(1.8%) | 5(8.9%) | 0(0.0%) | 4(7.1%) | 0(0.0%) | 12(21.4%) |
| 2000 | 19(33.9%) | 1(1.8%) | 9(16.1%) | 4(7.1%) | 0(0.0%) | 6(10.7%) | 0(0.0%) | 0(0.0%) | 1(1.8%) | 16(28.6%) |
| 2001 | 17(25.0%) | 5(7.4%) | 7(10.3%) | 3(4.4%) | 1(1.5%) | 14(20.6%) | 0(0.0%) | 3(4.4%) | 1(1.5%) | 17(25.0%) |
| 2002 | 41(38.7%) | 4(3.8%) | 10(9.4%) | 7(6.6%) | 0(0.0%) | 11(10.4%) | 0(0.0%) | 2(1.9%) | 0(0.0%) | 31(29.2%) |
| 2003 | 25(31.6%) | 2(2.5%) | 9(11.4%) | 6(7.6%) | 0(0.0%) | 6(7.6%) | 1(1.3%) | 3(3.8%) | 1(1.3%) | 26(32.9%) |
| 2004 | 32(47.8%) | 1(1.5%) | 9(13.4%) | 4(6.0%) | 0(0.0%) | 7(10.4%) | 0(0.0%) | 0(0.0%) | 1(1.5%) | 13(19.4%) |
| 2005 | 35(46.1%) | 3(3.9%) | 9(11.8%) | 1(1.3%) | 0(0.0%) | 9(11.8%) | 0(0.0%) | 2(2.6%) | 0(0.0%) | 17(22.4%) |
| 2006 | 36(49.3%) | 2(2.7%) | 7(9.6%) | 4(5.5%) | 1(1.4%) | 9(12.3%) | 0(0.0%) | 1(1.4%) | 1(1.4%) | 12(16.4%) |
| 2007 | 35(46.1%) | 3(3.9%) | 4(5.3%) | 5(6.6%) | 0(0.0%) | 4(5.3%) | 0(0.0%) | 2(2.6%) | 2(2.6%) | 21(27.6%) |
| 2008 | 42(53.2%) | 0(0.0%) | 6(7.6%) | 2(2.5%) | 0(0.0%) | 6(7.6%) | 0(0.0%) | 3(3.8%) | 2(2.5%) | 18(22.8%) |
| 2009 | 47(57.3%) | 0(0.0%) | 4(4.9%) | 3(3.7%) | 1(1.2%) | 9(11.0%) | 0(0.0%) | 2(2.4%) | 0(0.0%) | 16(19.5%) |
| 2010 | 70(64.2%) | 3(2.8%) | 5(4.6%) | 2(1.8%) | 1(0.9%) | 11(10.1%) | 1(0.9%) | 4(3.7%) | 0(0.0%) | 12(11.0%) |
| 2011 | 60(70.6%) | 2(2.4%) | 6(7.1%) | 2(2.4%) | 1(1.2%) | 2(2.4%) | 1(1.2%) | 4(4.7%) | 0(0.0%) | 7(8.2%) |
| 2012 | 70(68.6%) | 0(0.0%) | 7(6.9%) | 4(3.9%) | 1(1.0%) | 4(3.9%) | 1(1.0%) | 0(0.0%) | 0(0.0%) | 15(14.7%) |
| 2013 | 55(77.5%) | 0(0.0%) | 0(0.0%) | 2(2.8%) | 0(0.0%) | 6(8.5%) | 1(1.4%) | 0(0.0%) | 0(0.0%) | 7(9.9%) |
| 2014 | 66(77.6%) | 0(0.0%) | 0(0.0%) | 3(3.5%) | 0(0.0%) | 4(4.7%) | 0(0.0%) | 0(0.0%) | 0(0.0%) | 12(14.1%) |
| 2015 | 80(82.5%) | 1(1.0%) | 2(2.1%) | 3(3.1%) | 0(0.0%) | 5(5.2%) | 2(2.1%) | 1(1%) | 0(0.0%) | 3(3.1%) |
| 2016 | 84(90.3%) | 1(1.1%) | 1(1.1%) | 0(0.0%) | 0(0.0%) | 4(4.3%) | 0(0.0%) | 0(0.0%) | 1(1.1%) | 2(2.2%) |
| 2017 | 86(95.6%) | 0(0.0%) | 0(0.0%) | 0(0.0%) | 0(0.0%) | 1(1.1%) | 0(0.0%) | 0(0.0%) | 0(0.0%) | 3(3.3%) |
| 2018 | 72(96.0%) | 0(0.0%) | 0(0.0%) | 1(1.3%) | 0(0.0%) | 2(2.7%) | 0(0.0%) | 0(0.0%) | 0(0.0%) | 0(0.0%) |
| Total | 1047(53.6%) | 76(3.9%) | 125(6.4%) | 82(4.2%) | 8(0.4%) | 225(11.5%) | 9(0.5%) | 42(2.2%) | 14(0.7%) | 325(16.6%) |
