# Supplementary material for: Historical trends in histological composition and cause specific mortality of small intestine tumors based on SEER database analysis
Source: Sci Rep. 2025 May 28;15:18628. doi: 10.1038/s41598-025-03046-z (PMC12120026; doi:10.1038/s41598-025-03046-z)
Supplement: Supplementary file 6 — Supplementary Material 6 [file 41598_2025_3046_MOESM6_ESM.docx]

**Supplement Table 6 Age-stratified prevalence and outcomes of small intestinal tumor patients**

|  | ＜50 | 50-59 | 60-69 | 70-79 | ≥80 |
| --- | --- | --- | --- | --- | --- |
| Alive | 1768(64.6%) | 2338(58.6%) | 2498(48.8%) | 1526(32.5%) | 395(23.4%) |
| Small intestine | 313(11.4%) | 494(12.4%) | 706(13.8%) | 779(16.6%) | 318(18.9%) |
| Digestive tract | 172(6.3%) | 271(6.8%) | 371(7.2%) | 434(9.2%) | 197(11.7%) |
| Heart disease | 32(1.2%) | 95(2.4%) | 232(4.5%) | 376(8.0%) | 188(11.2%) |
| COPD | 4(0.1%) | 8(0.2%) | 32(0.6%) | 50(1.1%) | 31(1.8%) |
| Soft tissue | 69(2.5%) | 64(1.6%) | 79(1.5%) | 73(1.6%) | 10(0.6%) |
| Pancreas | 33(1.2%) | 52(1.3%) | 104(2.0%) | 147(3.1%) | 48(2.8%) |
| Miscellaneous malignant cancer | 120(4.4%) | 229(5.7%) | 323(6.3%) | 299(6.4%) | 102(6.0%) |
| Cerebrovascular disease | 8(0.3%) | 20(0.5%) | 39(0.8%) | 92(2.0%) | 39(2.3%) |
| Other causes of death | 219(8.0%) | 418(10.5%) | 736(14.4%) | 925(19.7%) | 358(21.2%) |
