# Supplementary material for: Historical trends in histological composition and cause specific mortality of small intestine tumors based on SEER database analysis
Source: Sci Rep. 2025 May 28;15:18628. doi: 10.1038/s41598-025-03046-z (PMC12120026; doi:10.1038/s41598-025-03046-z)
Supplement: Supplementary file 7 — Supplementary Material 7 [file 41598_2025_3046_MOESM7_ESM.docx]

**Supplement Table 7 Cox regression analysis of risk factors for mortality in patients with small intestinal tumors**

|  | HR | 95%CI | P |
| --- | --- | --- | --- |
| Age |  |  |  |
| <50 | 1.000 |  |  |
| 50-59 | 1.386 | 1.280-1.500 | <0.001 |
| 60-69 | 1.990 | 1.847-2.144 | <0.001 |
| 70-79 | 2.891 | 2.686-3.111 | <0.001 |
| ≥80 | 3.914 | 3.590-4.268 | <0.001 |
| Sex |  |  |  |
| Male | 1.000 |  |  |
| Female | 0.886 | 0.851-0.922 | <0.001 |
| Ethnicity |  |  |  |
| White | 1.000 |  |  |
| Black | 1.200 | 1.135-1.268 | <0.001 |
| Other | 1.036 | 0.959-1.118 | 0.374 |
| Primary site |  |  |  |
| Duodenum | 1.000 |  |  |
| Jejunum | 0.984 | 0.918-1.056 | 0.656 |
| lleum | 0.885 | 0.835-0.938 | <0.001 |
| Meckels diverticulum | 0.783 | 0.602-1.019 | 0.069 |
| Overlapping lesion | 1.034 | 0.851-1.256 | 0.735 |
| Small intestine, NOS | 1.024 | 0.968-1.083 | 0.406 |
| Grade |  |  |  |
| I, well differentiated | 1.000 |  |  |
| II, moderately differentiated | 1.812 | 1.683-1.951 | <0.001 |
| III, poorly differentiated | 2.565 | 2.366-2.781 | <0.001 |
| IV, undifferentiated or anaplastic | 2.549 | 2.227-2.918 | <0.001 |
| Unknown | 1.240 | 1.159-1.326 | <0.001 |
| Surgery |  |  |  |
| Yes | 1.000 |  |  |
| No | 2.269 | 2.143-2.402 | <0.001 |
| Unknown | 1.485 | 1.408-1.567 | <0.001 |
| Radiation |  |  |  |
| Yes | 1.000 |  |  |
| No/unknown | 0.879 | 0.804-0.960 | 0.004 |
| Chemotherapy |  |  |  |
| Yes | 1.000 |  |  |
| No/unknown | 0.637 | 0.606-0.671 | <0.001 |
| Bone metastases |  |  |  |
| Yes | 1.000 |  |  |
| No | 0.614 | 0.477-0.791 | <0.001 |
| Unknown | 1.030 | 0.513-2.065 | 0.935 |
| Liver metastases |  |  |  |
| Yes | 1.000 |  |  |
| No | 0.705 | 0.641-0.776 | <0.001 |
| Unknown | 0.967 | 0.657-1.423 | 0.865 |
| Lung metastases |  |  |  |
| Yes | 1.000 |  |  |
| No | 0.642 | 0.537-0.768 | <0.001 |
| Unknown | 0.670 | 0.435-1.032 | 0.069 |
| Brain metastases |  |  |  |
| Yes | 1.000 |  |  |
| No | 0.859 | 0.516-1.429 | 0.558 |
| Unknown | 0.526 | 0.213-1.302 | 0.165 |
