# Supplementary material for: Historical trends in histological composition and cause specific mortality of small intestine tumors based on SEER database analysis
Source: Sci Rep. 2025 May 28;15:18628. doi: 10.1038/s41598-025-03046-z (PMC12120026; doi:10.1038/s41598-025-03046-z)
Supplement: Supplementary file 10 — Supplementary Material 10 [file 41598_2025_3046_MOESM10_ESM.docx]

**Figure Legend**

**Supplement Figure 1** Specific mortality trends of small intestinal cancer and digestive tract cancer. (A) Specific mortality trends of small intestinal cancer compared to digestive tract cancer. (B) Specific mortality trends of small intestinal cancer and digestive tract cancer in carcinoid tumors. (C) Specific mortality trends of small intestinal cancer and digestive tract cancer in adenocarcinoma.

**Supplement Figure 2** The number of cardiovascular-specific deaths across all age groups from 1992 to 2018.

**Supplement Table 1** The change of constituent ratio for each histological type of small intestinal tumors from 1992 to 2018.

**Supplement Table 2** Cause-specific death in patients with small intestinal tumors of carcinoid tumor.

**Supplement Table 3** Cause-specific death in patients with small intestinal tumors of adenocarcinoma.

**Supplement Table 4** Cause-specific death in patients with small intestinal tumors of neuroendocrine carcinoma.

**Supplement Table 5** Cause-specific death in patients with small intestinal tumors of stromal sarcoma/leiomyosarcoma.

**Supplement Table 6** Age-stratified prevalence and outcomes of small intestinal tumor patients.

**Supplement Table 7** Cox regression analysis of risk factors for mortality in patients with small intestinal tumors.
